# Health, psychosocial, and economic impacts of the COVID-19 pandemic on people with chronic conditions in India: a mixed methods study

**DOI:** 10.1186/s12889-021-10708-w

**Published:** 2021-04-08

**Authors:** Kavita Singh, Dimple Kondal, Sailesh Mohan, Suganthi Jaganathan, Mohan Deepa, Nikhil Srinivasapura Venkateshmurthy, Prashant Jarhyan, Ranjit Mohan Anjana, K. M. Venkat Narayan, Viswanathan Mohan, Nikhil Tandon, Mohammed K. Ali, Dorairaj Prabhakaran, Karen Eggleston

**Affiliations:** 1 Public Health Foundation of India, Plot number 47, Sector 44, Gurugram, New Delhi, Haryana 122002 India; 2 Centre for Chronic Disease Control, New Delhi, India; 3 Deakin University, Melbourne, Australia; 4 Madras Diabetes Research Foundation, Chennai, India; 5 Emory University, Atlanta, GA USA; 6 All India Institute of Medical Sciences, New Delhi, India; 7 London School of Hygiene and Tropical Medicine, London, UK; 8 Stanford University, Stanford, California USA

**Keywords:** SARS coronavirus, COVID-19 pandemic, Chronic conditions, India

## Abstract

**Background:**

People with chronic conditions are disproportionately prone to be affected by the COVID-19 pandemic but there are limited data documenting this. We aimed to assess the health, psychosocial and economic impacts of the COVID-19 pandemic on people with chronic conditions in India.

**Methods:**

Between July 29, to September 12, 2020, we telephonically surveyed adults (*n* = 2335) with chronic conditions across four sites in India. Data on participants’ demographic, socio-economic status, comorbidities, access to health care, treatment satisfaction, self-care behaviors, employment, and income were collected using pre-tested questionnaires. We performed multivariable logistic regression analysis to examine the factors associated with difficulty in accessing medicines and worsening of diabetes or hypertension symptoms. Further, a diverse sample of 40 participants completed qualitative interviews that focused on eliciting patient’s experiences during the COVID-19 lockdowns and data analyzed using thematic analysis.

**Results:**

One thousand seven hundred thirty-four individuals completed the survey (response rate = 74%). The mean (SD) age of respondents was 57.8 years (11.3) and 50% were men. During the COVID-19 lockdowns in India, 83% of participants reported difficulty in accessing healthcare, 17% faced difficulties in accessing medicines, 59% reported loss of income, 38% lost jobs, and 28% reduced fruit and vegetable consumption. In the final-adjusted regression model, rural residence (OR, 95%CI: 4.01,2.90–5.53), having diabetes (2.42, 1.81–3.25) and hypertension (1.70,1.27–2.27), and loss of income (2.30,1.62–3.26) were significantly associated with difficulty in accessing medicines. Further, difficulties in accessing medicines (3.67,2.52–5.35), and job loss (1.90,1.25–2.89) were associated with worsening of diabetes or hypertension symptoms. Qualitative data suggest most participants experienced psychosocial distress due to loss of job or income and had difficulties in accessing in-patient services.

**Conclusion:**

People with chronic conditions, particularly among poor, rural, and marginalized populations, have experienced difficulties in accessing healthcare and been severely affected both socially and financially by the COVID-19 pandemic.

**Supplementary Information:**

The online version contains supplementary material available at 10.1186/s12889-021-10708-w.

## Background

As the global burden of novel coronavirus disease 2019 (COVID-19) continues to increase, particularly in low- and middle- income countries such as India, it imposes huge costs on individuals, communities, health systems, and economies [1]. Although some countries and regions are seeing improvements in hospitalization and death rates, COVID-19 remains a major concern for vulnerable and underserved populations globally [2, 3]. People with chronic conditions are disproportionately prone to COVID-19–related hospitalizations, intensive care admissions, and mortality, compared to those without chronic conditions [4–7]. Moreover, they may be particularly susceptible to adverse health impacts from delayed or foregone care during the pandemic. The 2020 World Health Organization (WHO) report on the impact of COVID-19 on noncommunicable diseases (NCDs) in 163 countries highlighted that nearly half of the countries report that patients experienced partial or complete disruption of services for hypertension, diabetes, and related complications during the pandemic. One-third reported disrupted services for cardiovascular emergencies. Further, most countries reassigned the health staff towards COVID-19 support, which affected routine care for NCDs [8]. Several reports indicated change in routine care to virtual consultations and worsened mental health problems during the pandemic. Diabetes, chronic obstructive pulmonary disease, and hypertension were the most impacted conditions due to significant reduction in access to care [9–14]. Given the syndemic interaction ─interrelationship between COVID-19 and various socio-ecological and biological factors contributing to preexisting NCD epidemics─ people with chronic conditions are more vulnerable to COVID-19 infection [15, 16].

As of March 11, 2021 more than 11 million people in India had been infected with COVID-19, and about 158,000 had died [6]. The spread of COVID-19 in India is of great concern due to the country’s large and densely populated areas with widespread poverty and high migration rates, coupled with a high prevalence of chronic conditions [17–19] that are generally poorly controlled [20–22]. Further, the progression of COVID-19 from urban to rural areas, the strict lockdown measures, and the associated economic shocks are likely to impede efforts to address other health scourges in India such as diabetes, hypertension, and cardiovascular diseases. On March 24, 2020, the Indian government ordered a nationwide lockdown, which was extended until June in four phases, and later further extended to specific containment zones. During the lockdowns, many health facilities were functioning sub-optimally or were converted to COVID facilities and provided only essential and emergency services.

Measures to address coronavirus spread including lockdowns may have serious economic consequences and unintended effect of exacerbating rather than mitigating health disparities [8, 9, 23, 24]. However, to date, few data document the impact of the COVID-19 pandemic on disparities in chronic disease management in India. Given the unprecedented and rapidly evolving COVID-19 situation in India, we aimed to assess the health, psychosocial and economic impacts of COVID-19 pandemic on people with chronic conditions in India.

## Methods

### Study setting and participants

We conducted a cross-sectional study using sequential mixed methods design, comprising a quantitative survey and qualitative interviews to describe the impact of COVID-19 on the health, psychosocial, and economic well-being of people with chronic conditions in India. Adults with one or more chronic conditions (hypertension, diabetes mellitus, cardiovascular disease, or chronic kidney disease), from the two large existing cohorts (the Centre for Cardio-metabolic Risk Reduction in South Asia, CARRS [25]; and a comprehensive diabetes and hypertension prevention and management program in India-UDAY [26]) were invited to participate in this study. The CARRS and UDAY study protocols and main study results have been published previously [14, 15]. Briefly, CARRS enrolled 12,271 adults aged ≥20 years that were sampled to be representative of Delhi and Chennai in 2010–2011 and has followed them annually since. The UDAY study enrolled 12,243 adults in 2014–15 aged ≥30 years from rural and urban communities in Sonipat (Haryana), and Visakhapatnam (Vizag), Andhra Pradesh, India. For this study, we used stratified random sampling of participants with chronic conditions by age and sex. We randomly selected and approached around 600 participants at each of the four sites (Delhi, Chennai Haryana and Vizag) in India. Furthermore, a diverse sample of 40 participants stratified by age, sex, comorbidities, and urban/rural sites were purposively selected for the qualitative interview. This study was approved by the Institutional Ethics Committees of the Centre for Chronic Disease Control, New Delhi, India, and the Madras Diabetes Research Foundation, Chennai, India. All participants provided verbal consent to this study over the phone.

### Data collection

Between July 29 and September 12, 2020, we collected data on participants’ demographic, socio-economic status, comorbidities, access to healthcare, difficulty in accessing medicines due to financial and non-financial (COVID-19 related) reasons, and treatment satisfaction. Quantitative survey questionnaire and qualitative study interview guide were developed by the authors for this mixed-methods study (Supplementary file 1). Participants were asked if their diabetes or hypertension symptoms worsened after lockdown. In addition, health status was assessed using EQ. 5D-VAS [27], and anxiety assessed using a generalized anxiety disorder (GAD) questionnaire [28]. Data on self-monitoring of blood glucose, adherence to diet plan, changes in physical activity, fruits and vegetables consumption pre- and post-lockdowns, employment status, and household income were collected using pre-tested questionnaire. Centralized online training was provided to the field workers to administer the survey over the telephone. Survey data were captured using Commcare application. Qualitative interviews were conducted in participant’s local language by trained researchers (KS, SJ) and focused on eliciting patient’s views on the challenges posed by the COVID-19 lockdowns and their mitigation.

### Statistical analysis

We used a sequential mixed methods study design to guide our analytical approach [29, 30]. Data are reported by study site and presented as a number (proportion) for categorical variables (e.g. access to health facility; diagnosed or hospitalized with COVID-19, loss of job or income) and a mean (SD) for normally distributed continuous variables (e.g., age, body mass index, health status score). GAD score [31] was defined as 0–4 no anxiety, 5–9 mild anxiety, 10–14 moderate anxiety, 15–21 severe anxiety. We performed bivariable and multivariable logistic regression analyses to find the factors associated with difficulty in accessing medicines and worsening diabetes or hypertension symptoms. We constructed three logistic regression models for each outcome. For the outcome “difficulty in accessing medicines”, Model 1 included demographic variables (age, sex, education and income); Model 2 included demographic variables and chronic conditions (diabetes, hypertension, cardiovascular disease and chronic kidney disease); Model 3, in addition to model 2 variables, included financial support from government (yes/no), loss of job (yes/no), and loss of income (yes/no) during the COVID-19 lockdowns. Next, for the outcome “worsening diabetes or hypertension symptoms”, Model 1 included demographic variables (age, sex, education and income); Model 2 included demographic variables and chronic conditions (diabetes, hypertension), GAD score (minimal, mild, moderate/severe), physical activity level, changes in fruit consumption during lockdown, and difficulty in accessing medicines (yes/no); Model 3, in addition to Model 2 variables, included financial support from the government (yes/no), loss of job (yes/no), and loss of income (yes/no) during the COVID-19 lockdowns. All data were analyzed using Stata version 16.0.

Qualitative data analyses focused on identifying views of individuals with chronic conditions, as well as the context, challenges, and mitigating factors or efforts to better manage chronic conditions during the COVID-19 pandemic in India. In-depth interviews with participants were audio-recorded, transcribed (verbatim), translated, anonymized, and checked for accuracy. The interview transcripts were coded thematically using MAXQDA software version 2020 [30]. Initial codes were developed and applied initially to a small number of transcripts, enabling further iteration of the thematic index [29, 32]. We used illustrative non-attributable quotations.

## Results

### Participant characteristics

Overall, 1734 out of 2335 contacted participants (74.3% response rate) completed the survey. We found 58 cohort members (2.5%) had died, 34 (1.5%) refused to participate, and 509 (22%) were not reachable for various reasons. Mean age (SD) of respondents was 57.8 (11.3) years, 50% were men, a majority had secondary school or college level education, and one-quarter of participants reported monthly household income of >INR 30,000 (Table 1). Most prevalent chronic conditions were hypertension (56%), diabetes (43%), and cardiovascular disease (13%). Of the participants surveyed, 3% were diagnosed or treated for COVID-19, 1% were hospitalized, and 69% reported that they had heard of a confirmed case of COVID-19 in their locality, more in urban (72%) than rural (58%) sites. About two-third of respondents experienced fear/anxiety related to COVID-19 and nearly half reported moderate difficulty in coping with stress during the COVID-19 lockdowns.

**Table 1:** Characteristics of study participants by demographic and socio-economic status, clinical history and COVID-19 status

	Overall (***N*** = 1734)	Delhi (***N*** = 430)	Chennai (***N*** = 494)	Sonipat (***N*** = 410)		Vizag (***N*** = 400)		***p***-value*
				Rural (***N*** = 209)	Urban (***N*** = 201)	Rural (***N*** = 192)	Urban (***N*** = 208)	
Age, years (mean, SD)	57.8 (11.3)	57.8 (11.0)	57.5 (11.6)	56.6 (11.4)	58.5 (10.8)	57.1 (10.2)	59.8 (11.9)	0.055
Male (%)	861 (49.7%)	219 (50.9%)	247 (50.0%)	86 (41.1%)	97 (48.3%)	92 (47.9%)	120 (57.7%)	0.033
Ownership of mobile phone	1366 (78.8%)	398 (92.6%)	382 (77.3%)	118 (56.5%)	123 (61.2%)	152 (79.2%)	193 (92.8%)	< 0.001
Internet use/access	741 (42.7%)	341 (79.3%)	175 (35.4%)	16 (7.7%)	50 (24.9%)	34 (17.7%)	125 (60.1%)	< 0.001
**Education**								
Below primary school	293 (16.9%)	43 (10.0%)	36 (7.3%)	57 (27.3%)	41 (20.4%)	93 (48.4%)	23 (11.1%)	< 0.001
Primary school	119 (6.9%)	9 (2.1%)	40 (8.1%)	10 (4.8%)	18 (9.0%)	28 (14.6%)	14 (6.7%)	
Secondary school	978 (56.4%)	219 (50.9%)	372 (75.3%)	135 (64.6%)	97 (48.3%)	65 (33.9%)	90 (43.3%)	
College degree and above	342 (19.7%)	157 (36.5%)	46 (9.3%)	7 (3.3%)	45 (22.4%)	6 (3.1%)	81 (38.9%)	
**Household income**								
< 10,000 INR	540 (31.1%)	51 (11.9%)	235 (47.6%)	96 (45.9%)	32 (15.9%)	115 (59.9%)	11 (5.3%)	< 0.001
10,000–30,000 INR	732 (42.2%)	169 (39.3%)	238 (48.2%)	77 (36.8%)	97 (48.3%)	64 (33.3%)	87 (41.8%)	
> 30,000 INR	431 (24.9%)	208 (48.4%)	21 (4.3%)	29 (13.9%)	52 (25.9%)	12 (6.3%)	109 (52.4%)	
**Occupation**								
Not working	1004 (57.9%)	259 (60.2%)	273 (55.3%)	137 (65.6%)	129 (64.2%)	86 (44.8%)	120 (57.7%)	< 0.001
Semiskilled/Unskilled	262 (15.1%)	26 (6.0%)	127 (25.7%)	30 (14.4%)	23 (11.4%)	37 (19.3%)	19 (9.1%)	
Trained/Skilled	456 (26.3%)	139 (32.3%)	91 (18.4%)	42 (20.1%)	49 (24.4%)	69 (35.9%)	66 (31.7%)	
White collar	12 (0.7%)	6 (1.4%)	3 (0.6%)	0 (0.0%)	0 (0.0%)	0 (0.0%)	3 (1.4%)	
BMI (Kg/m^2^), mean (SD)	26.8 (5.1)	27.8 (5.1)	26.8 (4.9)	26.1 (5)	27.2 (5.4)	23.7 (4.4)	27 (4.3)	< 0.001
Diabetes mellitus^a^	743 (42.8%)	180 (41.9%)	255 (51.6%)	35 (16.7%)	88 (43.8%)	49 (25.5%)	136 (65.4%)	< 0.001
Hypertension^b^	975 (56.2%)	202 (47.0%)	210 (42.5%)	154 (73.7%)	104 (51.7%)	152 (79.2%)	153 (73.6%)	< 0.001
Cardiovascular disease	229 (13.2%)	47 (10.9%)	38 (7.7%)	57 (27.3%)	35 (17.4%)	20 (10.4%)	32 (15.4%)	< 0.001
Chronic kidney disease	43 (2.5%)	9 (2.1%)	6 (1.2%)	12 (5.7%)	2 (1.0%)	7 (3.6%)	7 (3.4%)	0.006
Chronic obstructive pulmonary disease	18 (1.0%)	4 (0.9%)	2 (0.4%)	2 (1.0%)	1 (0.5%)	4 (2.1%)	5 (2.4%)	0.14
**COVID-19 status and related fear/anxiety**								
Participant diagnosed or treated for COVID-19	46 (2.7%)	18 (4.2%)	6 (1.2%)	13 (6.2%)	5 (2.5%)	2 (1.0%)	2 (1.0%)	0.003
Hospitalized for COVID-19	13 (0.7%)	4 (0.9%)	6 (1.2%)	0 (0.0%)	1 (0.5%)	1 (0.5%)	1 (0.5%)	< 0.001
Number of days hospitalized, median (IQR)	10.0 (8.0, 14.0)	13.0 (8.0, 14.0)	8.5 (8.0, 10.0)	na	10.0 (10.0, 10.0)	14.0 (14.0, 14.0)	14.0 (14.0, 14.0)	0.44
Immediate family members diagnosed/treated for COVID-19	52 (3.0%)	16 (3.7%)	13 (2.6%)	11 (5.3%)	8 (4.0%)	1 (0.5%)	3 (1.4%)	0.13
Any confirmed cases of COVID-19 in other households in your locality	1192 (68.7%)	265 (61.6%)	401 (81.2%)	111 (53.1%)	110 (54.7%)	123 (64.1%)	182 (87.5%)	< 0.001
Experienced fear/anxiety related to COVID-19	1029 (59.3%)	267 (62.1%)	148 (30.0%)	208 (99.5%)	131 (65.2%)	134 (69.8%)	141 (67.8%)	< 0.001
Experienced stigma related to COVID-19	15 (0.9%)	8 (1.9%)	2 (0.4%)	4 (1.9%)	1 (0.5%)	0 (0.0%)	0 (0.0%)	0.17
**Coping with stress during the COVID-19 situation**								
Very well	254 (14.6%)	77 (17.9%)	68 (13.8%)	44 (21.1%)	37 (18.4%)	6 (3.1%)	22 (10.6%)	< 0.001
Moderate	850 (49.0%)	190 (44.2%)	160 (32.4%)	164 (78.5%)	158 (78.6%)	92 (47.9%)	86 (41.3%)	
With difficulty	281 (16.2%)	93 (21.6%)	40 (8.1%)	0 (0.0%)	4 (2.0%)	70 (36.5%)	74 (35.6%)	
No Stress	349 (20.1%)	70 (16.3%)	226 (45.7%)	1 (0.5%)	2 (1.0%)	24 (12.5%)	26 (12.5%)	

*BMI* Body mass Index, *IQR* interquartile range (p25, p75), *INR* Indian rupees, *SD* standard deviation, *COVID-19* coronavirus disease 2019

*p value reported for between group difference across study sites

^a^Diabetes is defined based on fasting plasma glucose (FPG) > =126 mg/dl (7.0 mmol/l) and/or glycated haemoglobin (HbA1c) > = 6.5% (48 mmol/mol) or self-reported or on anti-diabetic medications. ^b^Hypertension was defined as being on antihypertensive medications or a systolic blood pressure > =140 mmHg and/or a diastolic blood pressure > =90 mmHg. Cardiovascular disease and chronic kidney disease were self-reported and/or on medications

### Rural versus urban comparison

**Fig. 1:**
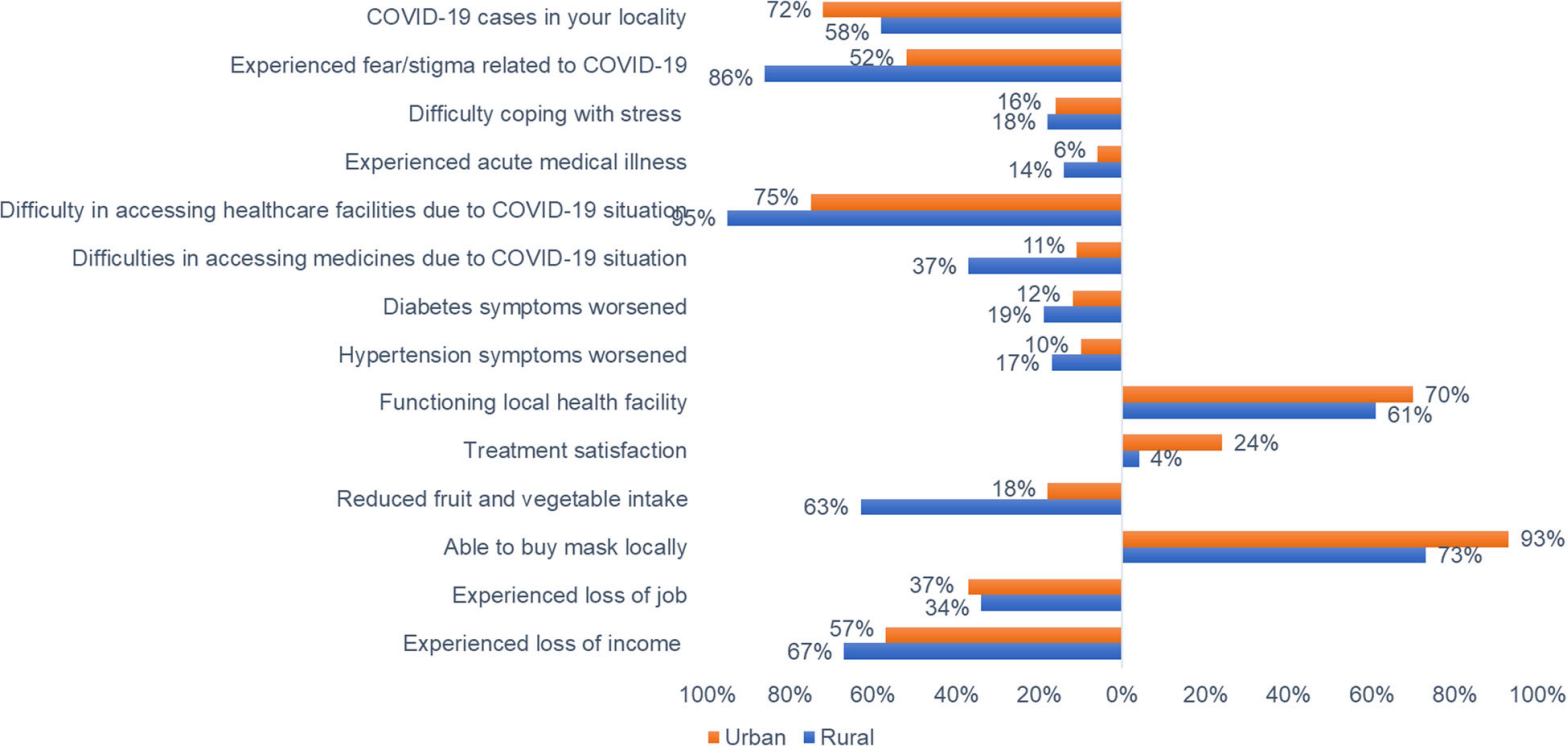
COVID-19 pandemic impacts on urban and rural people living with chronic conditions in India

Rural participants were disproportionately affected by the COVID-19 lockdowns compared with urban participants (Fig. 1). A greater proportion of rural participants experienced acute medical illness (rural 14.2%; urban 6.4%), difficulties in accessing health facilities (rural 95.0%; urban 75.0%) and medicines (rural 36.9%; urban 10.9%), worsened diabetes or hypertension symptoms (rural 16.0%; urban 11.0%), a lower treatment satisfaction rate (rural 3.5%; urban 23.8%), reduced fruit or vegetable consumption (rural 68.8%, urban 28.7%), and loss of household income (rural 67.3%, urban 56.9%).

### Health impacts

Across the four sites, 8% of study participants experienced an acute medical illness during the COVID-19 related lockdowns (Table 2) with higher proportions being affected in rural sites (14%). Two-thirds of patients reported that a local health clinic/hospital was functional during the COVID-19 lockdowns. Overall, the average health status score on EQ-VAS was 76.1; this was significantly lower in rural Vizag, 71.1. Nearly half the participants with diabetes or hypertension had their fasting blood sugar (FBS) or blood pressure (BP) tested during the lockdowns. Uncontrolled diabetes (FBS > 200 mg/dl) was reported by 19.3% of participants and uncontrolled systolic BP (> 140-160 mmHg) by 15.7%. About one-third of respondents perceived their blood sugar to be controlled and 15% perceived their BP to be under control.

**Table 2:** Impact of COVID-19 pandemic on health, healthcare access, treatment satisfaction and achievement of care goals

	Overall (***N*** = 1734)	Delhi (***N*** = 430)	Chennai (***N*** = 494)	Sonipat (***N*** = 410)		Vizag (***N*** = 400)		***p***-value*
				Rural (***N*** = 209)	Urban (***N*** = 201)	Rural (***N*** = 192)	Urban (***N*** = 208)	
Experienced acute medical illness during the COVID-19 lockdown	142 (8.2%)	25 (5.8%)	25 (5.1%)	31 (14.8%)	11 (5.5%)	26 (13.5%)	24 (11.5%)	< 0.001
Difficulty in accessing healthcare facility during COVID-19 lockdown	118 (83.1)	20 (80)	16 (64)	31 (100)	8 (72.7)	23 (88.5)	20 (83.3)	0.014
Experienced difficulties in accessing medicines due to COVID-19 situation	293 (16.9%)	27 (6.3%)	47 (9.5%)	69 (33.0%)	15 (7.5%)	79 (41.1%)	56 (26.9%)	
Diabetes mellitus	134 (18.0%)	17 (9.4%)	36 (14.1%)	11 (31.4%)	7 (8.0%)	24 (49.0%)	39 (28.7%)	0.24
Hypertension	198 (20.3%)	13 (6.4%)	20 (9.5%)	53 (34.4%)	11 (10.6%)	60 (39.5%)	41 (26.8%)	< 0.001
Cardiovascular disease	35 (15.3%)	1 (2%)	7 (18%)	16 (28%)	0 (0%)	4 (20%)	7 (22%)	0.002
Chronic kidney disease	7 (16%)	2 (22%)	1 (17%)	3 (25%)	0 (0%)	0 (0%)	1 (14%)	0.59
COPD	2 (11%)	0 (0%)	1 (50%)	1 (50%)	0 (0%)	0 (0%)	0 (0%)	0.22
Experienced difficulties in accessing medicines or treatment due to financial reasons	258 (14.9%)	16 (3.7%)	58 (11.7%)	36 (17.2%)	10 (5.0%)	88 (45.8%)	50 (24.0%)	
Diabetes mellitus	124 (16.7%)	12 (6.7%)	44 (17.3%)	7 (20.0%)	4 (4.5%)	23 (46.9%)	34 (25.0%)	0.75
Hypertension	162 (16.6%)	7 (3.5%)	28 (13.3%)	21 (13.6%)	7 (6.7%)	70 (46.1%)	29 (19.0%)	0.004
Cardiovascular disease	25 (10.9%)	0 (0%)	3 (8%)	9 (16%)	0 (0%)	5 (25%)	8 (25%)	0.10
Chronic kidney disease	4 (9%)	1 (11%)	0 (0%)	2 (17%)	0 (0%)	0 (0%)	1 (14%)	0.52
Chronic obstructive pulmonary lung disease	2 (11%)	0 (0%)	0 (0%)	1 (50%)	0 (0%)	0 (0%)	1 (20%)	
Functioning local health clinic/hospital during lockdown	1175 (67.8%)	329 (76.5%)	337 (68.2%)	153 (73.2%)	159 (79.1%)	91 (47.4%)	106 (51.0%)	< 0.001
Treatment satisfaction during lockdown	331 (19.1%)	96 (22.3%)	181 (36.6%)	3 (1.4%)	24 (11.9%)	11 (5.7%)	16 (7.7%)	
**Generalized anxiety disorder scale**								
Minimal anxiety	1497 (86.3%)	403 (93.7%)	451 (91.3%)	196 (93.8%)	194 (96.5%)	115 (59.9%)	138 (66.3%)	< 0.001
Mild anxiety	181 (10.4%)	25 (5.8%)	38 (7.7%)	11 (5.3%)	7 (3.5%)	60 (31.3%)	40 (19.2%)	
Moderate anxiety	34 (2.0%)	1 (0.2%)	4 (0.8%)	1 (0.5%)	0 (0.0%)	10 (5.2%)	18 (8.7%)	
Severe anxiety	22 (1.3%)	1 (0.2%)	1 (0.2%)	1 (0.5%)	0 (0.0%)	7 (3.6%)	12 (5.8%)	
**Overall health status score (EQ-VAS), mean (SD)**	76.1 (15.3) (n = 1734)	77.1 (16.5)	78.4 (15.7)	78.4 (15.6)	72.7 (16.3)	71.1 (11.1)	74.0 (11.7)	< 0.001
Mobility (moderate/severe problems)	360 (20.8%)	91 (21.2%)	78 (15.8%)	60 (28.7%)	58 (28.9%)	45 (23.4%)	28 (13.5%)	
Self-care (moderate/severe problems)	136 (7.8%)	23 (5.3%)	41 (8.3%)	16 (7.7%)	29 (14.4%)	14 (7.3%)	13 (6.3%)	
Usual care (moderate/severe problems)	223 (12.9%)	31 (7.2%)	59 (11.9%)	38 (18.2%)	39 (19.4%)	29 (15.1%)	27 (13.0%)	
Pain/discomfort (moderate/severe problems)	448 (25.8%)	135 (31.4%)	70 (14.2%)	79 (37.8%)	70 (34.8%)	56 (29.2%)	38 (18.3%)	
Anxiety/depression (moderate/severe problems)	314 (18.1%)	58 (13.5%)	83 (16.8%)	46 (22.0%)	41 (20.4%)	51 (26.6%)	35 (16.8%)	
**Health consequences in people with diabetes (N)**	**743**	**180**	**255**	**35**	**88**	**49**	**136**	
Fasting blood sugar tested during the lockdown	414 (55.7%)	130 (72.2%)	151 (59.2%)	13 (37.1%)	37 (42.0%)	17 (34.7%)	66 (48.5%)	< 0.001
HbA1c tested during the lockdown	35 (4.7%)	10 (5.6%)	8 (3.1%)	0 (0.0%)	4 (4.5%)	0 (0.0%)	13 (9.6%)	< 0.001
Fasting blood sugar, mean (SD)	166.6 (71.8)	158.0 (64.5)	185.9 (83.1)	287.3 (83.6)	137.4 (32.1)	197.1 (63.4)	139.7 (44.2)	< 0.001
Fasting blood sugar > 160–200 mg/dl	51 (17.9)	19 (16.2)	18 (19.6)	1 (16.7)	3 (30)	3 (42.9)	7 (13.2)	
Fasting blood sugar > 200 mg/dl	55 (19.3)	17 (14.5)	29 (31.5)	5 (83.3)	0 (0)	2 (28.6)	2 (3.8)	
Blood sugar controlled (perceived)	230 (31.0%)	35 (19.4%)	87 (34.1%)	16 (45.7%)	19 (21.6%)	27 (55.1%)	46 (33.8%)	< 0.001
Diabetes symptoms worsened during the lockdown	97 (13.1%)	33 (18.3%)	23 (9.0%)	8 (22.9%)	9 (10.2%)	8 (16.3%)	16 (11.8%)	< 0.001
Glucose monitoring frequency at home								
Once in month	83 (11.2%)	43 (23.9%)	13 (5.1%)	2 (5.7%)	16 (18.2%)	1 (2.0%)	8 (5.9%)	
Do not monitor glucose at home	530 (71.3%)	70 (38.9%)	223 (87.5%)	33 (94.3%)	62 (70.5%)	46 (93.9%)	96 (70.6%)	
**Health consequences in people with hypertension (N)**	**975**	**202**	**210**	**154**	**104**	**152**	**153**	
Blood pressure measured during the lockdown	515 (52.8%)	137 (67.8%)	127 (60.5%)	70 (45.5%)	39 (37.5%)	53 (34.9%)	89 (58.2%)	< 0.001
Systolic blood pressure, mean (SD)	139.0 (20.3)	142.5 (18.8)	135.4 (19.4)	152.2 (29.7)	140.3 (19.2)	133.3 (19.5)	132.2 (14.2)	< 0.001
Diastolic blood pressure, mean (SD)	86.9 (13.5)	88.7 (11.1)	84.6 (11.0)	91.8 (25.9)	85.8 (17.0)	84.8 (11.3)	84.6 (7.1)	0.042
SBP ≤140 mmHg	271 (74.6%)	79 (69.9%)	38 (67.9%)	22 (56.4%)	27 (75.0%)	25 (86.2%)	80 (88.9%)	0.008
SBP > 140–160 mmHg	57 (15.7%)	19 (16.8%)	12 (21.4%)	9 (23.1%)	6 (16.7%)	3 (10.3%)	8 (8.9%)	
SBP > 160 mmHg	35 (9.6%)	15 (13.3%)	6 (10.7%)	8 (20.5%)	3 (8.3%)	1 (3.4%)	2 (2.2%)	
Blood pressure controlled (perceived)	142 (14.6%)	18 (8.9%)	65 (31.0%)	24 (15.6%)	5 (4.8%)	21 (13.8%)	9 (5.9%)	0.022
Symptoms of hypertension worsened during the lockdown	120 (12.3%)	42 (20.8%)	6 (2.9%)	38 (24.7%)	8 (7.7%)	13 (8.6%)	13 (8.5%)	< 0.001

Diabetes is defined based on fasting plasma glucose (FPG) > =126 mg/dl (7.0 mmol/l) and/or glycated haemoglobin (HbA1c) > = 6.5% (48 mmol/mol) or self-reported or on anti-diabetic medications. Hypertension was defined as being on antihypertensive medications or a systolic blood pressure > =140 mmHg and/or a diastolic blood pressure > =90 mmHg. Cardiovascular disease and chronic kidney disease were self-reported and/or on medications

*HbA1c* glycated haemoglobin, *COPD* chronic obstructive pulmonary lung disease, *EQ-VAS* European Quality of Life 5-dimension, Visual analogue scale, *SBP* systolic blood pressure, *DBP* diastolic blood pressure, *SD* standard deviation, *INR* Indian rupees, *COVID-19* coronavirus disease 2019, *mmHg* millimoles of mercury

*p value reported for between group difference across study sites

**Fig. 2:**
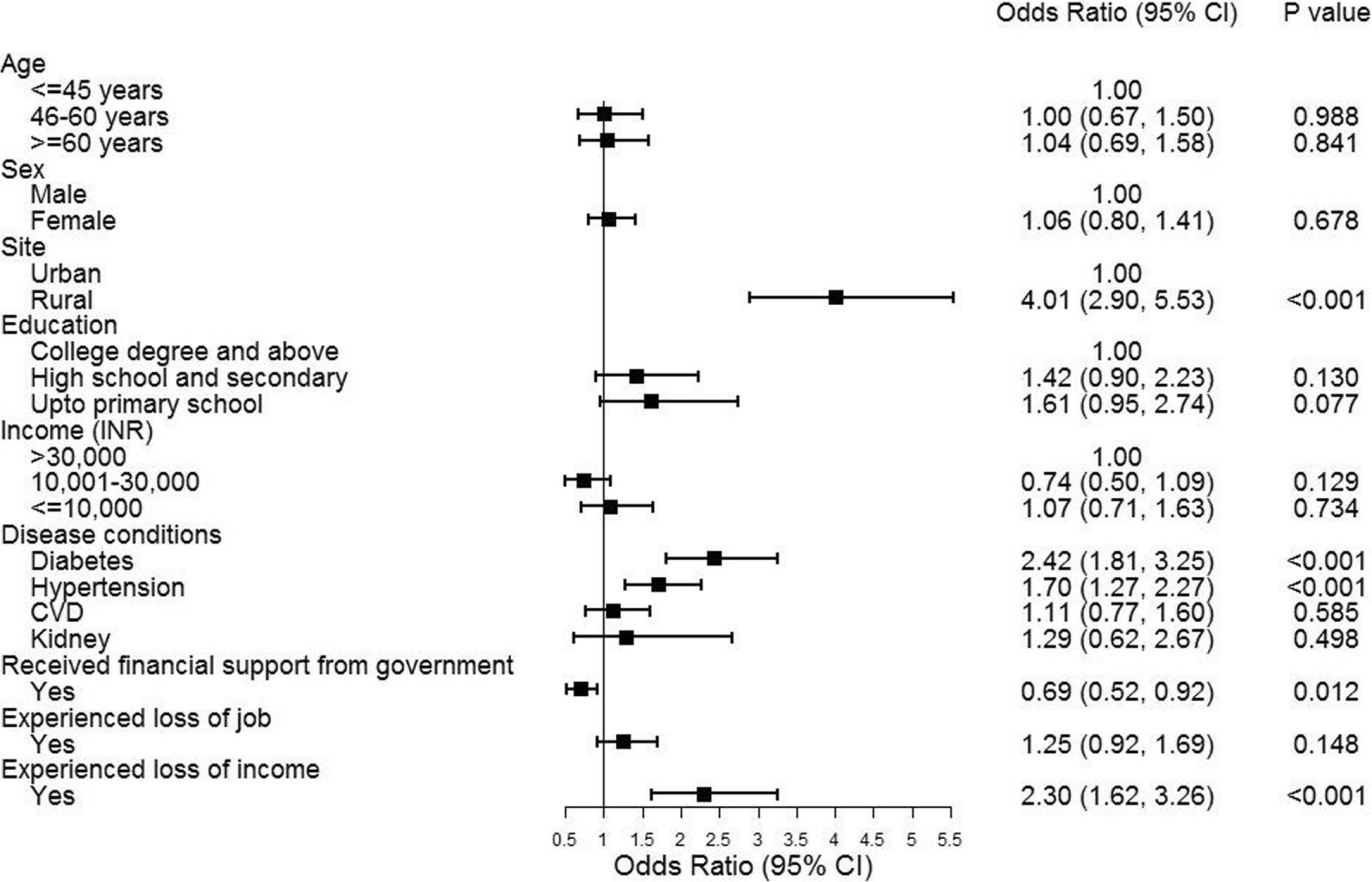
Factors associated with difficulty in accessing medicines due to the COVID-19 situation. *Diabetes is defined based on fasting plasma glucose (FPG) > =126 mg/dl (7.0 mmol/l) and/or glycated haemoglobin (HbA1c) > = 6.5% (48 mmol/mol) or self-reported or on anti-diabetic medications. Hypertension was defined as being on antihypertensive medications or a systolic blood pressure > =140 mmHg and/or a diastolic blood pressure > =90 mmHg. Cardiovascular disease and chronic kidney disease were self-reported and/or on medications. INR = Indian rupees, CVD = cardiovascular disease, Kidney = chronic kidney disease, 95% CI = confidence interval*

**Fig. 3:**
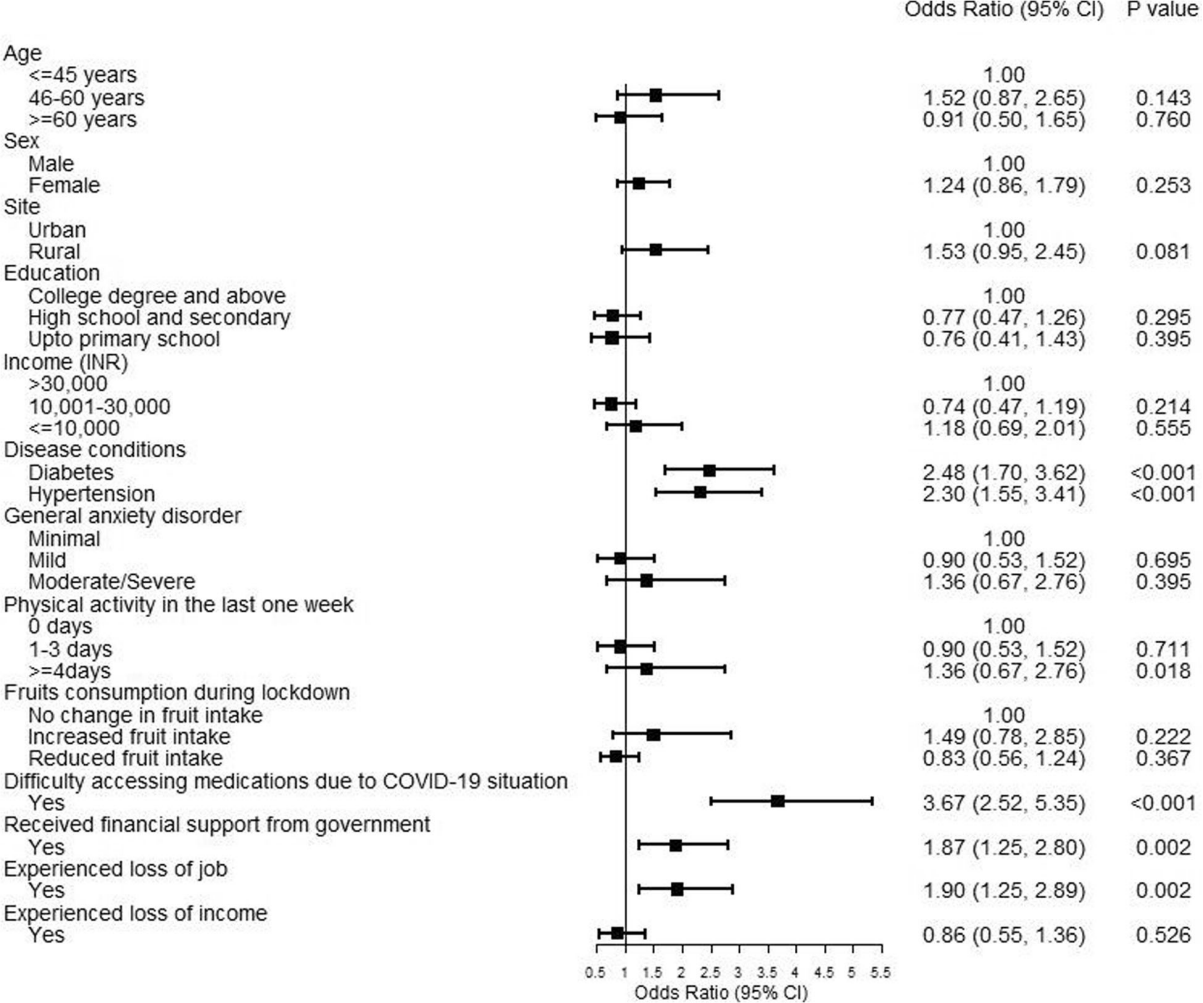
Factors associated with worsening of diabetes or hypertension symptoms during the COVID-19 lockdown. *Diabetes is defined based on fasting plasma glucose (FPG) > =126 mg/dl (7.0 mmol/l) and/or glycated haemoglobin (HbA1c) > = 6.5% (48 mmol/mol) or self-reported or on anti-diabetic medications. Hypertension was defined as being on antihypertensive medications or a systolic blood pressure > =140 mmHg and/or a diastolic blood pressure > =90 mmHg. INR = Indian rupees, COVID-19 = coronavirus disease 2019, 95% CI = confidence interval*

In the final-adjusted multivariable regression model, we found rural participants (odds ratio (OR), 95% confidence interval (CI): 4.01,2.90–5.53), having diabetes (2.42,1.81–3.25) and hypertension (1.70,1.27–2.27), and loss of income (2.30,1.62–3.26) were significantly associated with difficulty in accessing medicines. Financial aid from the government reduced the odds of difficulty in accessing medicines, i.e., had protective effect (OR: 0.69, 95%CI:0.52–0.92) (Fig. 2 and online Table S1). Figure 3 and online Table S2 demonstrate the factors associated with worsening of diabetes or hypertension symptoms. In the regression Model 1, adjusted for demographic and socio-economic variables, we found rural participants and females had higher odds of worsening diabetes or hypertension symptoms compared with urban or male counterparts (OR, 95%CI: 1.53,1.07–2.21 and 1.49,1.08–2.06, respectively). However, in the full multivariable-adjusted regression model, we found difficulties in accessing medicines (3.67, 2.52–5.35), loss of job (1.90, 1.25–2.89), and financial support from the government (1.87, 1.25–2.80) to be significantly associated with worsening of diabetes or hypertension symptoms.

### Psychosocial and economic impacts

One-third of respondents did not adhere to their recommended diet plan and reduced fruit and vegetable consumption during the lockdowns (Table 3). About two-third of respondents did not perform physical activity and reported loss of household income, and one-third had lost jobs. Overall, 45% of participants had received financial support from the government, with large variation by site (93% in Chennai vs. 8% in Delhi).

**Table 3:** Social and economic consequences of COVID-19 pandemic

	Overall (***N*** = 1734)	Delhi (***N*** = 430)	Chennai (***N*** = 494)	Sonipat (***N*** = 410)		Vizag (***N*** = 400)	
Impact on self-care behaviors				Rural (***N*** = 209)	Urban (***N*** = 201)	Rural (***N*** = 192)	Urban (***N*** = 208)
Adherence to a meal plan in the last 1 week							
0 days	553 (36.2)	179 (51.6)	227 (54.7)	48 (25)	46 (25)	37 (20)	16 (7.8)
1–3 days	157 (10.3)	4 (1.2)	0 (0)	52 (27.1)	57 (31)	26 (14.1)	18 (8.7)
4 or more days	819 (53.6)	164 (47.3)	188 (45.3)	92 (47.9)	81 (44)	122 (66)	172 (83.5)
Irregular eating pattern in the last 1 week							
0 days	1365 (78.7)	388 (90.2)	456 (92.3)	126 (60.3)	116 (57.7)	134 (69.8)	145 (69.7)
1–3 days	249 (14.4)	32 (7.4)	25 (5.1)	39 (18.7)	60 (29.9)	44 (22.9)	49 (23.6)
4 or more days	120 (6.9)	10 (2.3)	13 (2.6)	44 (21.1)	25 (12.4)	14 (7.3)	14 (6.7)
Physical activity in the last 1 week							
0 days	1038 (59.9)	332 (77.2)	338 (68.4)	54 (25.8)	90 (44.8)	122 (63.5)	102 (49)
1–3 days	202 (11.7)	36 (8.4)	12 (2.4)	83 (39.7)	54 (26.9)	7 (3.7)	10 (4.8)
4 or more days	494 (28.5)	62 (14.4)	144 (29.2)	72 (34.5)	57 (28.4)	63 (32.8)	96 (46.2)
Fruits consumption during lockdown vs. pre-lockdown							
Reduced fruit intake	658 (37.9%)	77 (17.9%)	153 (31.0%)	198 (94.7%)	94 (46.8%)	78 (40.6%)	58 (27.9%)
Increased fruit intake	105 (6.1%)	40 (9.3%)	10 (2.0%)	1 (0.5%)	5 (2.5%)	13 (6.8%)	36 (17.3%)
No change in fruit intake	971 (56.0%)	313 (72.8%)	331 (67.0%)	10 (4.8%)	102 (50.7%)	101 (52.6%)	114 (54.8%)
Vegetables consumption during lockdown vs. pre-lockdown							
Reduced vegetable intake	485 (28.0%)	49 (11.4%)	67 (13.6%)	191 (91.4%)	81 (40.3%)	61 (31.8%)	36 (17.3%)
Increased vegetable intake	196 (11.3%)	46 (10.7%)	39 (7.9%)	2 (1.0%)	8 (4.0%)	35 (18.2%)	66 (31.7%)
No change in vegetable intake	1053 (60.7%)	335 (77.9%)	388 (78.5%)	16 (7.7%)	112 (55.7%)	96 (50.0%)	106 (51.0%)
Duration and intensity of physical activity during lockdown vs. pre-lockdown							
Decreased physical activity	431 (24.9%)	42 (9.8%)	111 (22.5%)	149 (71.3%)	89 (44.3%)	10 (5.2%)	30 (14.4%)
Increased physical activity	38 (2.2%)	10 (2.3%)	11 (2.2%)	2 (1.0%)	8 (4.0%)	0 (0.0%)	7 (3.4%)
No change in physical activity	227 (13.1%)	46 (10.7%)	34 (6.9%)	4 (1.9%)	14 (7.0%)	60 (31.3%)	69 (33.2%)
**Economic impact of COVID-19**							
Experienced loss of job	634 (36.6%)	163 (37.9%)	212 (42.9%)	86 (41.1%)	75 (37.3%)	49 (25.5%)	49 (23.6%)
Experienced loss of income	1029 (59.3%)	203 (47.2%)	355 (71.9%)	131 (62.7%)	91 (45.3%)	139 (72.4%)	110 (52.9%)
Received financial support from the government	774 (44.6%)	36 (8.4%)	460 (93.1%)	34 (16.3%)	14 (7.0%)	163 (84.9%)	67 (32.2%)

*COVID-19* coronavirus disease 2019

Because of the pandemic and related restrictions imposed to reduce its spread, few participants (15.2%) reported visiting friends/family, although about half were able to leave their locality to buy food or other supplies (52.6%). The majority reported that fruits, vegetables, and essential groceries were available during the lockdowns (96.8%), although we do not know prices or quality. Most participants (99.4%) reported being aware of and following recommended preventive measures such as wearing mask, handwashing, and social distancing (online Table S3).

### Qualitative study results

Our sample consisted of 40 participants with one or more chronic conditions, mean age: 54.6 years, and 64% were men. Thematic redundancy was achieved with 8th interview, and two participants were then interviewed from each of the four sites (total, *N* = 40) to confirm thematic redundancy. Interviews lasted about 20–40 min. Two major themes emerged from qualitative data analysis: “challenges faced”, and “resilience and mitigating factors”.

### Challenges faced

Most participants faced financial difficulties during the COVID-19 lockdowns. Several participants reported difficulty getting to work because of lack of public transportation. Some participants lost their jobs due to the COVID-19 pandemic, as reflected in the following quotes from study participants:

*“We faced difficulties at home because I am into driving. Before lockdown, I went home for some work. Because of lockdown, I had to stay at home for 2.5 months. I, my wife, and children are jobless since then. There was no possibility of doing any work or going anywhere. We had a lot of trouble at that time.” (R-02-V)*

*“The impact was that there were a lot of problems. We took the ration distributed by the government. We consumed that. There were a few things [at home], we sold one or two things with the help of my daughter. My son drives a rickshaw, and my husband stays at home; we are old. It impacted him [spouse]. He was out of work for three months.” (U-10-D)*

Some participants had difficulty accessing inpatient services, since many hospitals were full or refused new admissions due to COVID-19 cases. Many participants were concerned about visiting the hospital or doctor and delayed testing of their blood sugar because of fear and anxiety about COVID-19.

*“I was not keeping well and none of the hospitals were taking any admission . . . they [hospital staff] said that due to COVID, beds are not available. And if you are ready to sleep on ground then we will take your admission.” (U-08-V)*

*“I was scared that I may not have this [coronavirus infection] but because of someone else I may get affected. We have doubt to go to the hospital, to the doctor. I didn’t want to get infected by this (COVID-19).” (U-02-D)*

Participants with diabetes and hypertension were almost all aware of their elevated risk of poor outcomes if infected with SARS-CoV-2 and many feared to go out for a walk or other regular exercise.

### Resilience and mitigating factors

Participants were well informed and emphasized the importance of wearing masks, practicing social distancing, or handwashing. Few participants utilized teleconsultations with doctors to avoid making in-person clinic visits. Most participants embraced the practice of enhanced personal cleanliness and other measures to proactively reduce risks of COVID-19 infection and transmission.

*“We have to be careful from the corona and we have to be safe from this. That’s the only medicine now.” (U-01-D)*

## Discussion

COVID-19 pandemic related restrictions implemented to control it had unforeseen adverse impacts on the health status, access to treatment, and achievement of care goals among people with chronic conditions in India. We found rural participants disproportionately experienced acute medical illnesses; difficulties in accessing healthcare; relatively less availability of functioning health facilities; poor treatment satisfaction; and reduced fruit and vegetable consumption.

Infectious disease epidemics have tended to have spillover effects onto the wider economy [14, 33–37]. This study showed that impacts of the pandemic extend beyond health to encompass adverse effects on household incomes, individual livelihoods, interpersonal relationships, coping skills, nutritional intake, and other factors. Our quantitative and qualitative data underscore significant economic impacts from loss of employment and household income in the study population, due at least in part to restrictions preventing workers from returning to work. Those repercussions may in turn lead to further stress and additional impacts on health. People with diabetes and hypertension were worst affected due to their difficulty in accessing health care and experienced worsening symptoms or uncontrolled BP or FBS during the lockdowns, which might lead to poor health outcomes and avoidable micro- and macrovascular complications. People with chronic conditions are known to be most vulnerable to the complications of COVID-19 as highlighted in the WHO global survey and several published reports [8, 9, 23, 38–40]. It is unclear how the dual impact of COVID-19 and the health care disruptions affect these individuals in the long-term.

Our study results are consistent with other online surveys conducted among people with chronic conditions and healthcare providers that showed the coronavirus pandemic and its related lockdowns significantly reduced access to healthcare, adversely impacted self-care behaviors, and increased mental health problems [11, 15, 16, 24, 38]. A recent study from India reported the effects of COVID-19–related lockdowns on the adoption of newer technologies and changes in glycemic control in patients with diabetes and found that the pandemic did not poorly affect glycemic control (HbA1c levels before vs. during lockdown: 8.2% vs. 7.7%). However, that study was conducted at a single private clinic, and the higher socio-economic status of the surveyed participants could influence the study results [41]. Another cross-sectional study from India evaluating the impact of COVID-19 related lockdowns on changes in health behaviors and metabolic parameters in people with diabetes found that adherence to therapy, glycemic control, and monitoring did not differ significantly pre- and post-lockdowns [42]. However, in a sub-analysis of our study, we noted significant increase in the mean FBS reported during lockdown (198 mg/dl) vs. before lockdown (165 mg/dl) in the cohort participants. This indicates that people with diabetes appear to be at greater risk of experiencing uncontrolled blood sugar during the pandemic, which is consistent with the results of another study from India that found diabetes to be the most common comorbidity among COVID-19 decedents [43]. COVID-19 has also been a major concern in higher-income countries, with many European countries and the United States experiencing significant excess mortality in 2020 and a greater proportion of deaths from NCDs at home [44–49].

The COVID-19 pandemic is unprecedented and serious, and several of the policy measures taken to mitigate and contain it were necessary and understandable. At the same time, we believe that the data from our study provide insights for policy makers as they consider the asymmetrical psycho-social and economic impacts of the pandemic on people with chronic conditions, especially underprivileged urban residents and underserved rural communities. In our study, rural residents and those of lower educational attainment experienced more difficulties in accessing medicines, controlling for other demographics and self-reported income. Difficulty in access to medicines, in turn was associated with worsening of diabetes or hypertension symptoms. Global supply chain disruptions during the pandemic contributed to reported shortages of essential medicines for chronic conditions [50]. Furthermore, the pandemic caused people with chronic conditions to face many lifestyle disruptions (unhealthy diet and physical inactivity, sleep disturbances, stress, and anxiety) needing remedial measures [16, 51]. Government aid was associated with fewer difficulties in access to medicines, but varied significantly across locations, demonstrating the importance of appropriate policies at the state and local levels. To mitigate the disparities in chronic disease management and reduce the potential longer-run health impacts of the current crisis, a promising approach is to focus on enabling access to medicines for vulnerable populations (i.e., those in rural areas, with lower educational attainment, and those experiencing poverty exacerbated by loss of jobs and household income). New models of healthcare delivery combined with new skills (e.g., patient-centered orientation and leveraging consumer-facing technologies) for the health workforce can promote patient engagement and health literacy, ultimately improving health outcomes.

Our data may assist health authorities to redesign care delivery models to address the urgent needs of people with chronic conditions. We recommend a three-pronged approach to design resilient healthcare systems during and after the COVID-19 pandemic: a) develop and implement digital campaigns to disseminate information on how to adopt healthy behaviors, better self-manage NCDs, and control COVID-19; b) decentralize healthcare delivery for people with chronic conditions by involving trained community health workers and using technology-assisted medical interventions along with home monitoring devices to improve health care services; c) provide effective social and economic support for people with chronic conditions, particularly rural communities, elderly, and those with severe mental health problems. Many have experienced loss of livelihoods, isolation, stress, and anxiety during the pandemic; however, those with preexisting chronic conditions have often experienced compounding effects that exacerbate their illness [52]. Therefore, social networks and family members have an important role to play within the community and at home in monitoring and enhancing self-care behaviors among patients with chronic conditions. Although regulatory authorities in many countries have approved one or more COVID-19 vaccines for emergency use, important challenges remain in mass producing and distributing vaccines in developing countries. In addition, the lasting and complex syndemic effects of the pandemic may linger; therefore, social health measures remain important. Greater investment in prevention efforts and strengthening primary care can help save future healthcare costs, reduce the burden of NCDs, and enhance resilience against future pandemics [53].

The strength of this study lies in its empirical mixed methods study design and focus on people with chronic conditions from both urban and rural populations. It is the first such study from a populous country like India. However, future research is needed to evaluate the longer-run impact of the COVID-19 pandemic on healthcare access and health outcomes for those both with and without NCDs.

### Limitations

This study has important limitations. First, the cross-sectional nature of this study limits the causal inferences between SES and chronic conditions and the COVID-19 pandemic related restrictions. Second, although the data are derived from a wide cross section of four sites, it cannot be construed as definitively representative of all urban and rural India. Third, because of the ongoing COVID-19 outbreak that we are studying, it was not possible to conduct the interviews in person; phone interviews may have limited the interpretation of qualitative data since they do not allow direct observation of participants’ expressions and body language.

## Conclusion

In response to the rapid spread of the COVID-19 pandemic and associated health system disruptions experienced in under-resourced and low-income settings, there needs to be renewed focus on building resilient health systems that can deliver routine care using innovative telehealth approaches during the pandemic and respond to the shocks induced by infectious disease pandemics or other health crises effectively. People living in rural areas and underserved communities in urban areas faced greater challenges in access to healthcare and experienced worsening of diabetes or hypertension symptoms, as well as significant losses of income and employment. The pandemic exposed disparities in chronic disease management, but also provides opportunities to close gaps with innovations in the new post-COVID India.

## Supplementary Information

**Additional file 1: Table S1.** Factors associated with difficulty in accessing medicines during the COVID-19 lockdowns in India. **Table S2**. Factors associated with worsening of diabetes or hypertension symptoms during the COVID-19 lockdowns in India. **Table S3.** COVID-19 pandemic-related restrictions and preventive measures.

**Additional file 2.** Supplementary file.

## Data Availability

The datasets generated and analyzed during the current study are not publicly available because the datasets are currently used for another project but are available from the corresponding author on reasonable request.

## Abbreviations

BP: Blood pressure
CARRS: Centre for Cardiometabolic Risk Reduction in South Asia
CCDC: Centre for Chronic Disease Control
CI: Confidence Interval
COVID-19: Coronavirus disease 2019
EQ 5D-VAS: European Quality of Life 5 Dimension - Visual Analogue Scale
FBS: Fasting Blood Sugar
GAD: Generalized Anxiety Disorder
MDRF: Madras Diabetes Research Foundation
NCD: Non-Communicable Disease
OR: Odds Ratio
SARS: Severe Acute Respiratory Syndrome
SD: Standard Deviation
WHO: World Health Organization
