# Supplementary material for: Health, psychosocial, and economic impacts of the COVID-19 pandemic on people with chronic conditions in India: a mixed methods study
Source: BMC Public Health. 2021 Apr 8;21:685. doi: 10.1186/s12889-021-10708-w (PMC8027966; doi:10.1186/s12889-021-10708-w)
Supplement: Supplementary file 1 — Additional file 1: Table S1. Factors associated with difficulty in accessing medicines during the COVID-19 lockdowns in India. Table S2. Factors associated with worsening of diabetes or hypertension symptoms during the COVID-19 lockdowns in India. Table S3. COVID-19 pandemic-related restrictions and preventive measures. [file 12889_2021_10708_MOESM1_ESM.docx]

**Online Supplemental Tables**

**Table S1.** Factors associated with difficulty in accessing medicines during the COVID-19 lockdowns in India

**Table S2**. Factors associated with worsening of diabetes or hypertension symptoms during the COVID-19 lockdowns in India

**Table S3.** COVID-19 pandemic-related restrictions and preventive measures.

**Table S1**. Factors associated with difficulty in accessing medicines during the COVID-19 lockdowns in India.

|  | **Difficulty in accessing medicines** | | **Unadjusted** | **Model 1** | **Model 2** | **Model 3** |
| --- | --- | --- | --- | --- | --- | --- |
|  | **No (n=1254)** | **Yes (377)** | **Odds ratio (95% CI)** | **Odds ratio (95% CI)** | **Odds ratio (95% CI)** | **Odds ratio (95% CI)** |
| **Age** |  |  |  |  |  |  |
| ≤*45 years* | 181 (14.4%) | 53 (14.1%) | 1.00 | 1.00 | 1.00 | 1.00 |
| *46-60 years* | 561 (44.7%) | 174 (46.2%) | 1.06 (0.75,1.5) | 1.15 (0.79,1.67) | 1 (0.68,1.46) | 1.00 (0.67,1.5) |
| *≥60 years* | 512 (40.8%) | 150 (39.8%) | 1 (0.7,1.43) | 1.08 (0.74,1.59) | 0.91 (0.61,1.35) | 1.04 (0.69,1.58) |
| **Gender** |  |  |  |  |  |  |
| Male | 639 (51.0%) | 168 (44.6%) | 1.00 | 1.00 | 1.00 | 1.00 |
| *Female* | 615 (49.0%) | 209 (55.4%) | 1.29 (1.03,1.63) | 1.07 (0.82,1.39) | 1 (0.77,1.31) | 1.06 (0.8,1.41) |
| **Site** |  |  |  |  |  |  |
| *Urban* | 1025 (81.7%) | 205 (54.4%) | 1.00 | 1.00 | 1.00 | 1.00 |
| *Rural* | 229 (18.3%) | 172 (45.6%) | 3.76 (2.93,4.81) | 3 (2.29,3.94) | 3.69 (2.73,4.99) | 4.01 (2.9,5.53) |
| **Education** |  |  |  |  |  |  |
| *College degree and above* | 271 (21.6%) | 36 (9.5%) | 1.00 | 1.00 | 1.00 | 1.00 |
| *High school and secondary* | 714 (57.0%) | 208 (55.2%) | 2.19 (1.5,3.21) | 1.59 (1.05,2.4) | 1.62 (1.07,2.48) | 1.42 (0.9,2.23) |
| *Up to primary school* | 267 (21.3%) | 133 (35.3%) | 3.75 (2.5,5.62) | 1.87 (1.15,3.04) | 1.94 (1.18,3.18) | 1.61 (0.95,2.74) |
| **Income (INR)** |  |  |  |  |  |  |
| *>30,000* | 346 (28.2%) | 170 (45.8%) | 1.00 | 1.00 | 1.00 | 1.00 |
| *10,001-30,000* | 552 (44.9%) | 136 (36.7%) | 1.25 (0.91,1.74) | 0.96 (0.68,1.36) | 0.96 (0.68,1.37) | 0.74 (0.5,1.09) |
| *≤10,000* | 331 (26.9%) | 65 (17.5%) | 2.5 (1.81,3.46) | 1.44 (1,2.07) | 1.5 (1.03,2.18) | 1.07 (0.71,1.63) |
| **Disease conditions^** |  |  |  |  |  |  |
| *Diabetes* | 556 (44.3%) | 187 (49.6%) | 1.24 (0.98,1.56) |  | 2.33 (1.76,3.08) | 2.42 (1.81,3.25) |
| *Hypertension* | 714 (56.9%) | 261 (69.2%) | 1.7 (1.33,2.18) |  | 1.6 (1.21,2.1) | 1.70 (1.27,2.27) |
| *Cardiovascular disease* | 173 (13.8%) | 56 (14.9%) | 1.09 (0.79,1.51) |  | 1.06 (0.75,1.5) | 1.11 (0.77,1.6) |
| *Chronic kidney disease* | 31 (2.5%) | 12 (3.2%) | 1.3 (0.66,2.55) |  | 1.22 (0.6,2.47) | 1.29 (0.62,2.67) |
| **Received financial support from government** | | | |  |  |  |
| *Yes* | 655 (52.2%) | 151 (40.1%) | 0.56 (0.44,0.71) |  |  | 0.69 (0.52,0.92) |
| **Experienced loss of job in the family during this pandemic** | | | |  |  |  |
| *Yes* | 424 (33.8%) | 174 (46.2%) | 1.69 (1.34,2.14) |  |  | 1.25 (0.92,1.69) |
| **Experienced loss of household income during this pandemic** | | | |  |  |  |
| *Yes* | 682 (54.4%) | 293 (77.7%) | 3.02 (2.29,3.97) |  |  | 2.30 (1.62,3.26) |

*^ Diabetes is defined based on fasting plasma glucose (FPG) >=126 mg/dl (7.0 mmol/l) and/or glycated hemoglobin (HbA1c) >= 6.5% (48 mmol/mol) or self-reported or on anti-diabetic medications. Hypertension was defined as being on antihypertensive medications or a systolic blood pressure >=140 mmHg and/or a diastolic blood pressure >=90 mmHg. Cardiovascular disease and chronic kidney disease were self-reported and/or on medications. INR=Indian rupees.*

***Model 1*** *(multivariable logistic regression analysis) included demographic variables (age, sex, education and income);* ***Model 2*** *included demographic variables and chronic conditions (diabetes, hypertension, cardiovascular and chronic kidney disease);* ***Model 3****, in addition to model 2 variables, included financial support from government (yes/no), loss of job (yes/no), and loss of income (yes/no) during the COVID-19 lockdowns.*

**Table S2**. Factors associated with worsening of diabetes or hypertension symptoms during the COVID-19 lockdowns in India.

|  | **Worsening of diabetes/ hypertension symptoms** | | **unadjusted** | **Model 1** | **Model 2** | **Model 3** |
| --- | --- | --- | --- | --- | --- | --- |
|  | **No (n=1429)** | **Yes (n=199)** | **Odds ratio (95% CI)** | **Odds ratio (95% CI)** | **Odds ratio (95% CI)** | **Odds ratio (95% CI)** |
| **Age** |  |  |  |  |  |  |
| ≤*45 years* | 212 (14.8%) | 21 (10.6%) | 1.00 | 1.00 | 1.00 | 1.00 |
| *46-60 years* | 631 (44.2%) | 103 (51.8%) | 1.65 (1.01,2.7) | 1.72 (1.04, 2.85) | 1.46 (0.86,2.48) | 1.52 (0.87,2.65) |
| *≥60 years* | 586 (41.0%) | 75 (37.7%) | 1.29 (0.78,2.15) | 1.32 (0.78, 2.23) | 0.95 (0.54,1.66) | 0.91 (0.5,1.65) |
| **Gender** |  |  |  |  |  |  |
| Male | 725 (50.7%) | 81 (40.7%) | 1.00 | 1.00 | 1.00 | 1.00 |
| *Female* | 704 (49.3%) | 118 (59.3%) | 1.5 (1.11,2.03) | 1.49 (1.08, 2.06) | 1.22 (0.86,1.73) | 1.24 (0.86,1.79) |
| **Site** |  |  |  |  |  |  |
| *Urban* | 1092 (76.4%) | 135 (67.8%) | 1.00 | 1.00 | 1.00 | 1.00 |
| *Rural* | 337 (23.6%) | 64 (32.2%) | 1.54 (1.11,2.12) | 1.53 (1.07, 2.21) | 1.29 (0.82,2.03) | 1.53 (0.95,2.45) |
| **Education** |  |  |  |  |  |  |
| *College degree and above* | 270 (18.9%) | 36 (18.2%) | 1.00 | 1.00 | 1.00 | 1.00 |
| *High school and secondary* | 814 (57.0%) | 106 (53.5%) | 0.98 (0.65,1.46) | 0.87 (0.56, 1.35) | 0.8 (0.5,1.28) | 0.77 (0.47,1.26) |
| *Up to primary school* | 344 (24.1%) | 56 (28.3%) | 1.22 (0.78,1.91) | 0.83 (0.48, 1.45) | 0.75 (0.41,1.36) | 0.76 (0.41,1.43) |
| **Income (INR)** |  |  |  |  |  |  |
| *>30,000* | 340 (24.3%) | 54 (27.7%) | 1.00 | 1.00 | 1.00 | 1.00 |
| *10,001-30,000* | 620 (44.2%) | 68 (34.9%) | 0.69 (0.47,1.01) | 0.67 (0.45, 1.01) | 0.68 (0.45,1.05) | 0.74 (0.47,1.19) |
| ≤*10,000* | 442 (31.5%) | 73 (37.4%) | 1.04 (0.71,1.52) | 0.94 (0.61, 1.45) | 0.94 (0.58,1.5) | 1.18 (0.69,2.01) |
| **Disease conditions^** |  |  |  |  |  |  |
| *Diabetes* | 629 (44.0%) | 114 (57.3%) | 1.71 (1.26,2.3) |  | 2.17 (1.52,3.1) | 2.48 (1.7,3.62) |
| *Hypertension* | 827 (57.9%) | 148 (74.4%) | 2.11 (1.51,2.95) |  | 2.15 (1.49,3.12) | 2.30 (1.55,3.41) |
| **General Anxiety disorder (GAD)** | |  |  |  |  |  |
| *Minimal* | 1243 (87.0%) | 155 (77.9%) | 1.00 |  | 1.00 | 1.00 |
| *Mild* | 147 (10.3%) | 29 (14.6%) | 1.58 (1.03,2.44) |  | 1.01 (0.63,1.64) | 0.90 (0.53,1.52) |
| *Moderate/Severe* | 39 (2.7%) | 15 (7.5%) | 3.08 (1.66,5.72) |  | 1.5 (0.75,2.97) | 1.36 (0.67,2.76) |
| **Performance of moderate physical activity for more than 10 minutes in the last one week** | | | | |  |  |
| 0 days | 1120 (78.4%) | 143 (71.9%) | 1.00 |  | 1.00 | 1.00 |
| 1-3 days | 205 (14.3%) | 42 (21.1%) | 1.32 (0.87,2.02) |  | 1.57 (0.96,2.57) | 0.90 (0.53,1.52) |
| ≥4days | 104 (7.3%) | 14 (7.0%) | 0.64 (0.44,0.92) |  | 0.63 (0.41,0.94) | 1.36 (0.67,2.76) |
| **During lockdown quantity of fruits consumed compared to pre lockdown period** | | | | |  |  |
| No change in fruit intake | 803 (56.2%) | 102 (51.3%) | 1.00 |  | 1.00 | 1.00 |
| Increased fruit intake | 77 (5.4%) | 17 (8.5%) | 1.74 (0.99,3.06) |  | 1.33 (0.72,2.46) | 1.49 (0.78,2.85) |
| Reduced fruit intake | 549 (38.4%) | 80 (40.2%) | 1.15 (0.84,1.57) |  | 0.86 (0.59,1.26) | 0.83 (0.56,1.24) |
| **Difficulty in accessing medicines due to COVID-19 situation or financial reasons** | | | | |  |  |
| Yes | 280 (19.6%) | 97 (48.7%) | 3.90 (2.87,5.31) |  | 3.49 (2.45,4.97) | 3.67 (2.52,5.35) |
| **Received some financial support from government** | | | |  |  |  |
| *Yes* | 692 (50.4%) | 114 (60.0%) | 1.47 (1.08,2.01) |  |  | 1.87 (1.25, 2.80) |
| **Experienced loss of job during this pandemic in the family** | | | |  |  |  |
| *Yes* | 502 (35.1%) | 95 (47.7%) | 1.71 (1.26,2.31) |  |  | 1.90 (1.25,2.89) |
| **Experienced loss of income during this pandemic in the family** | | | |  |  |  |
| *Yes* | 845 (59.1%) | 129 (64.8%) | 1.32 (0.96,1.81) |  |  | 0.86 (0.55,1.36) |

*^ Diabetes is defined based on fasting plasma glucose (FPG) >=126 mg/dl (7.0 mmol/l) and/or glycated hemoglobin (HbA1c) >= 6.5% (48 mmol/mol) or self-reported or on anti-diabetic medications. Hypertension was defined as being on antihypertensive medications or a systolic blood pressure >=140 mmHg and/or a diastolic blood pressure >=90 mmHg. INR=Indian rupees.*

***Model 1*** *(multivariable logistic regression analysis) included demographic variables (age, sex, education and income);* ***Model 2*** *included demographic variables and chronic conditions (diabetes, hypertension, cardiovascular and chronic kidney disease), a GAD score (minimal, mild, moderate/severe), physical activity level, changes in fruit consumption during lockdown, and difficulty accessing medicines (yes/no);* ***Model 3****, in addition to model 2 variables, included financial support from the government (yes/no), loss of job (yes/no), and loss of income (yes/no) during the COVID-19 lockdowns.*

**Table S3.** COVID-19 pandemic related restrictions and preventive measures.

|  | **Overall**  **(N=1734)** | **Delhi**  **(N=430)** | **Chennai**  **(N=494)** | **Sonipat (N=410)** | | **Vizag (N=400)** | |
| --- | --- | --- | --- | --- | --- | --- | --- |
|  |  |  |  | **Rural (N=209)** | **Urban (N=201)** | **Rural (N=192)** | **Urban (N208)** |
| Visiting friends/family outside your locality | 264 (15.2%) | 88 (20.5%) | 76 (15.4%) | 2 (1.0%) | 16 (8.0%) | 42 (21.9%) | 40 (19.2%) |
| Ability to leave the locality to buy food or other supplies | 912 (52.6%) | 227 (52.8%) | 413 (83.6%) | 10 (4.8%) | 38 (18.9%) | 107 (55.7%) | 117 (56.3%) |
| Needed permission to leave the cities/villages/towns | 715 (41.2%) | 68 (15.8%) | 205 (41.5%) | 167 (79.9%) | 62 (30.8%) | 105 (54.7%) | 108 (51.9%) |
| Gather with your neighbors for activities | 66 (3.8%) | 7 (1.6%) | 29 (5.9%) | 10 (4.8%) | 2 (1.0%) | 10 (5.2%) | 8 (3.8%) |
| Visit neighbor’s home in the locality | 90 (5.2%) | 5 (1.2%) | 54 (10.9%) | 2 (1.0%) | 21 (10.4%) | 6 (3.1%) | 2 (1.0%) |
| Children in the locality/township allowed to freely play outside | 132 (7.6%) | 0 (0.0%) | 60 (12.1%) | 3 (1.4%) | 57 (28.4%) | 7 (3.6%) | 5 (2.4%) |
| Hand washing | 1726 (99.5%) | 429 (99.8%) | 491 (99.4%) | 207 (99.0%) | 200 (99.5%) | 191 (99.5%) | 208 (100.0%) |
| Social distancing | 1724 (99.4%) | 430 (100.0%) | 485 (98.2%) | 209 (100.0%) | 200 (99.5%) | 192 (100.0%) | 208 (100.0%) |
| Wearing mask | 1724 (99.4%) | 429 (99.8%) | 490 (99.2%) | 209 (100.0%) | 198 (98.5%) | 191 (99.5%) | 207 (99.5%) |
| Cleaning fruits and vegetables before use | 1680 (96.9%) | 429 (99.8%) | 446 (90.3%) | 208 (99.5%) | 200 (99.5%) | 190 (99.0%) | 207 (99.5%) |
| People required to wear masks when leaving their homes | 1720 (99.2%) | 424 (98.6%) | 490 (99.2%) | 208 (99.5%) | 200 (99.5%) | 190 (99.0%) | 208 (100.0%) |
| Able to buy any appropriate masks locally | 1532 (88.4%) | 379 (88.1%) | 490 (99.2%) | 171 (81.8%) | 172 (85.6%) | 122 (63.5%) | 198 (95.2%) |
| Use any appropriate mask while being outdoors | 1683 (97.1%) | 412 (95.8%) | 490 (99.2%) | 207 (99.0%) | 195 (97.0%) | 172 (89.6%) | 207 (99.5%) |
| Availability of fresh fruits/vegetables | 1679 (96.8%) | 412 (95.8%) | 491 (99.4%) | 209 (100.0%) | 190 (94.5%) | 170 (88.5%) | 207 (99.5%) |
| Availability of groceries/supplies (e.g., rice, flour, milk/dairy products) | 1703 (98.2%) | 414 (96.3%) | 493 (99.8%) | 209 (100.0%) | 197 (98.0%) | 183 (95.3%) | 207 (99.5%) |
| Availability of animal foods like eggs/meat/non-vegetarian foods | 1296 (74.7%) | 272 (63.3%) | 469 (94.9%) | 160 (76.6%) | 45 (22.4%) | 155 (80.7%) | 195 (93.8%) |
| Price of general living materials changed compared to last year or pre lockdown | | | | | |  |  |
| Increased | 1315 (75.8%) | 247 (57.4%) | 374 (75.7%) | 206 (98.6%) | 186 (92.5%) | 142 (74.0%) | 160 (76.9%) |
| Decreased | 26 (1.5%) | 6 (1.4%) | 1 (0.2%) | 0 (0.0%) | 1 (0.5%) | 16 (8.3%) | 2 (1.0%) |
| No change | 241 (13.9%) | 116 (27.0%) | 65 (13.2%) | 1 (0.5%) | 5 (2.5%) | 27 (14.1%) | 27 (13.0%) |
| Aware of anyone who had to leave the city for medical care | 217 (12.5%) | 13 (3.0%) | 15 (3.0%) | 39 (18.7%) | 27 (13.4%) | 65 (33.9%) | 58 (27.9%) |
