# Supplementary material for: Health, psychosocial, and economic impacts of the COVID-19 pandemic on people with chronic conditions in India: a mixed methods study
Source: BMC Public Health. 2021 Apr 8;21:685. doi: 10.1186/s12889-021-10708-w (PMC8027966; doi:10.1186/s12889-021-10708-w)
Supplement: Supplementary file 2 — Additional file 2. Supplementary file. [file 12889_2021_10708_MOESM2_ESM.pdf]

Supplementary file 1: Survey questionnaire

Study title: Impact of COVID-19 pandemic on individuals with chronic conditions in India

**Survey questionnaire**			**Date of Interview:**	
**Participant Name:**	**Cohort Name:**	**Place of residence:**	**Age: (in completed years)**	**Sex: M/F**
**Ownership of mobile phone: Yes/No**			**Internet use/access: Yes/No**	

1	Have you ever been told by the doctor that you have any of the following conditions?		
	Diabetes Mellitus	☐Yes	☐No
	Blood pressure	☐Yes	☐No
	Heart disease	☐Yes	☐No
	Kidney disease	☐Yes	☐No
	Chronic Obstructive Pulmonary Disease	☐Yes	☐No
2	During this COVID-19 related national lockdown, did you experience any acute medical illness or medical emergency? ☐Yes ☐No ☐Don't know		
3	How difficult was it to access healthcare facility for acute illness/emergency during lockdown?<br>☐Very difficult ☐Somewhat difficult ☐Neutral ☐Easy ☐Very easy		
4	Was the local health clinic/hospital functioning in your area/locality during lockdown?<br>☐Yes ☐No ☐Don't know		
5	How satisfied are you with your current access to treatment?<br>Very satisfied 6 [ ] 5 [ ] 4 [ ] 3 [ ] 2 [ ] 1 [ ] 0 [ ] Very dissatisfied		

**6.0**	**COVID-19 status in your family**<br>*(<u>Instructions for the data collector</u>: Explain briefly to the study participant, now I would like to ask a few questions about COVID-19 situation amongst your immediate family members (sons, daughters, parents) i.e. people residing in the same household)*			
6.1	Were you diagnosed or treated for COVID-19?	☐Yes	☐No	☐Don't know
6.2	If yes, were you hospitalized for COVID-19?	☐Yes	☐No	☐Don't know
6.3	If yes, how long?	[ ] [ ] number of days		
6.4	Were any of your immediate family members diagnosed with COVID-19?	☐Yes	☐No	☐Don't know
6.5	If yes, were they hospitalized?	☐Yes	☐No	☐Don't know
6.6	If yes, how long?	[ ] [ ] number of days		
6.7	Did anyone die of COVID-19 in your immediate family?	☐Yes	☐No	☐Don't know
6.6	If yes, number of deaths?	[ ] [ ]		
**7.0**	**Your current health status and impact of COVID-19 on chronic conditions (**Q. Nos. 7.13 - 7.18 only for patients with diabetes**)**			
7.1	Have you experienced difficulties accessing medication or treatment due to **COVID-19 situation** in the past month?<br><br>If yes, for which of the following?	☐Yes	☐No	☐Don't know
7.2-7.6	Diabetes Mellitus	☐Yes	☐No	
	Blood pressure	☐Yes	☐No	
	Heart disease	☐Yes	☐No	
	Kidney disease	☐Yes	☐No	

	Chronic obstructive pulmonary disease	☐Yes	☐No	
7.7	Have you experienced difficulties accessing medication or treatment due to **financial factors** in the past month? If yes, for which of the following?	☐Yes	☐No	☐Don't know
7.8-7.12	Diabetes Mellitus	☐Yes	☐No	
	Blood pressure	☐Yes	☐No	
	Heart disease	☐Yes	☐No	
	Kidney disease	☐Yes	☐No	
	Chronic Obstructive Pulmonary Disease	☐Yes	☐No	
7.13	How frequently do you monitor your glucose at home?<br>Once every day ☐ Once in two days ☐<br>Once weekly ☐ Twice weekly ☐<br>Twice a month ☐ Once a month ☐<br>I do not monitor glucose at home ☐			
7.14	Have you had your fasting blood sugar tested during the lockdown?	☐Yes	☐No	☐Don't know
7.15	Have you had your HbA1c tested during the lockdown?	☐Yes	☐No	☐Don't know
7.16	If yes, on what date?	FBS test date: ________<br>HbA1c test date: _________		
7.17	What was your fasting blood sugar / HbA1c? [If you do not know exact number, do you know if it was under control or out of control?]	FBS (mg/dL): ________________<br>HbA1c (%): ________________<br>Blood sugar controlled:<br>☐Yes ☐No ☐Don't know		
7.18	Have your symptoms of diabetes or high blood sugar worsened since your area/locality where you stay went on lockdown?	☐Yes	☐No	☐Don't know
7.19	Was your blood pressure measured during the lockdown at home or a clinic/hospital?	☐Yes	☐No	☐Don't know
7.20	If yes, on what date?			
7.21	What was your last blood pressure reading?<br>If you do not know can you tell if it was under control?	SBP (mmHg)_______<br>DBP (mmHg)_______<br>Blood pressure controlled:<br>☐Yes ☐No ☐Don't know		
7.22	Have your symptoms of hypertension or high blood pressure worsened since your area of residence went on lockdown?	☐Yes	☐No	☐Don't know
7.23	Have you experienced fear/stigma related to COVID-19 infection?	☐Yes	☐No	☐Don't know
7.24	How are you coping with stress during the current COVID-19 situation?<br>☐very well ☐moderate ☐with difficulty ☐not applicable (no stress)			
**8.0**	**COVID-19 status and restrictions in your locality**<br>*[In this section, I would like to ask you few questions on the COVID 19 situation in your locality, any restriction on movement of people and goods]*			
8.1	Have you heard of any confirmed cases of COVID-19 in other households in your locality?	☐Yes	☐No	☐Don't know
8.2-8.7	In general, during the lockdown period,<br>If someone from outside your locality (like family or friends) wanted to visit your locality, were they allowed to enter the locality during the lockdown?	☐Yes	☐No	☐Don't know

	Were you able to leave the locality to buy food or other supplies?	☐Yes	☐No	☐Don't know
	Did you need permission to leave the cities/villages/towns?	☐Yes	☐No	☐Don't know
	Were you able to gather with your neighbours for activities (e.g., morning walks/exercise/parties/festivals/religious practices/ weddings/funerals)?	☐Yes	☐No	☐Don't know
	Were you able to visit neighbour's home in the locality (e.g., for meals or to play cards)?	☐Yes	☐No	☐Don't know
	Are children in the locality/township allowed to freely play outside (for instance, in the streets)?	☐Yes	☐No	☐Don't know
8.8	Do you know if the following measures were recommended by local/state/national governments to control COVID-19 spread?	☐Yes	☐No	☐Don't know
	Hand washing	☐Yes	☐No	☐Don't know
	Social distancing	☐Yes	☐No	☐Don't know
	Wearing mask	☐Yes	☐No	☐Don't know
	Cleaning fruits and vegetables before use	☐Yes	☐No	☐Don't know
8.9	In general, are people required to wear masks when leaving their homes?	☐Yes	☐No	☐Don't know
**9.0**	**Availability of preventive measures and daily essentials**			
9.1	Are you able to buy any appropriate masks locally?	☐Yes	☐No	☐Don't know
9.2	Do you use any appropriate mask (surgical/home-made) while being outdoors?	☐Yes	☐No	☐Don't know
9.3-9.5	Currently, please let us know about the availability of the following items in your locality			
	Fresh fruits/vegetables	☐Yes	☐No	☐Don't know
	Groceries/supplies (e.g., rice, flour, and cooking oil)	☐Yes	☐No	☐Don't know
	Animal foods like eggs/meat/non-vegetarian foods	☐Yes	☐No	☐Don't know
9.6	How has the price of general living materials changed compared to last year or pre lockdown?	☐ Increased	☐ Decreased	☐No change<br>☐Don't know
9.7	Have you heard of anyone who needed to leave the city for medical care (for any reason) but cannot see a doctor right now?	☐Yes	☐No	☐Don't know
**10.0**	**Consequences of COVID-19 on employment and household income**			
10.1	What is your current employment status?	☐Yes	☐No	☐Don't know
10.2	Due to the lockdown, were you able to continue work?	☐Work from home<br>☐No work<br>☐Went to work less frequently		
10.3	Have you or members in your immediate family experienced a loss in job as a result of COVID-19?	☐Yes	☐No	☐Don't know
10.4	Have you or members in your immediate family experienced a loss in income as a result of COVID-19?	☐Yes	☐No	☐Don't know
10.5	Have you received any kind of financial support from the government? (applicable if there was loss of income, job or to those belonging to poor SES/ Jandhan account holders)	☐Yes	☐No	☐Don't know
**11.0**	**Your health status: EQ-5D –Place a tick in one box in each group below for the statement that best describes your own health state today**			
11.1	Mobility<br>1 ☐ I have no problems in walking about<br>2 ☐ I have some problems in walking about<br>3 ☐ I am confined to bed			
11.2	Self-Care<br>1 ☐ I have no problems with self-care<br>2 ☐ I have some problems with self-care<br>3 ☐ I am unable to wash or dress myself			

11.3	Usual Activities (e.g. work, study, housework, family or leisure activities)<br>1 ☐ I have no problems with performing my usual activities<br>2 ☐ I have some problems with performing my usual activities<br>3 ☐ I am unable to perform my usual activities
11.4	Pain/Discomfort<br>1 ☐ I have no pain or discomfort<br>2 ☐ I have moderate pain or discomfort<br>3 ☐ I have extreme pain or discomfort
11.5	Anxiety/Depression<br>1 ☐ I am not worried or depressed<br>2 ☐ I am moderately anxious or depressed<br>3 ☐ I am extremely anxious or depressed
11.6	Your health status on a scale of 0-100: ___ ___ ___
**12.0**	**Generalized Anxiety Disorder 7-item (GAD-7) scale**
12. 1	Over the last 2 weeks, how often have you been bothered by the following problems?

		Not at all sure	Several days	Over half the days	Nearly every day
12. 2	Feeling nervous, anxious, or on edge	0	1	2	3
12. 3	Not being able to stop or control worrying	0	1	2	3
12. 4	Worrying too much about different things	0	1	2	3
12. 5	Trouble relaxing	0	1	2	3
12. 6	Being so restless that it's hard to sit still	0	1	2	3
12. 7	Becoming easily annoyed or irritable	0	1	2	3
12. 8	Feeling afraid as if something awful might happen	0	1	2	3

12. 9	If you checked off any problems, how difficult have these made it for you to do your work, take care of things at home, or get along with other people?<br><br>☐ Not difficult at all ☐Somewhat difficult ☐ Very difficult ☐ Extremely difficult
**13. 0**	**Impact of COVID-19 on diet and physical activity**

13. 1	In the past one week, how many days did you adhere to a meal plan with low/restricted salt/sugar/fat?	☐ 0 days<br>☐ 1 day<br>☐ 2 days<br>☐ 3 days<br>☐ 4 or more days
13. 2	In the past one week on how many days did you have irregular eating pattern (e.g. change in meal time/ frequent snacking)?	☐ 0 days<br>☐ 1 day<br>☐ 2 days<br>☐ 3 days<br>☐ 4 or more days

13. 3	During the lockdown, did the quantity of fruits and vegetables consumed change compared to pre lockdown period?	☐Yes, I consumed less F&V<br>☐Yes, I consumed more F&V<br>☐No, I consumed the same amount of F&V
13. 4	In the past one week, on how many days were you able to perform moderate physical activity for more than 10 minutes?	☐ 0 days<br>☐ 1 day<br>☐ 2 days<br>☐ 3 days<br>☐ 4 or more days
13. 5	During the lockdown did the duration and intensity of physical activity change compared to pre-lockdown period?	☐Yes, I performed less physical activity<br>☐Yes, I performed more physical activity<br>☐No, I performed same duration and intensity of physical activity

Qualitative Study Interview Guide — Patient version 1.0; Date: 01 June 2020

1. Please can you describe how long you have been diagnosed with and/or taking treatment for your chronic condition(s): diabetes/hypertension/CVD/CKD/COPD?
2. What are the greatest challenges that you are facing due to the COVID19 outbreak and lockdown?
    a. Please describe what changes/adjustments have you brought into your routine life due to the COVID19 outbreak?
        i. How do you protect yourself from the COVID19?
    b. How the COVID19 situation has impacted on managing your chronic condition, in terms of:
        i. lifestyle behaviours/self-care goals: diet, exercise, stress/depression, tobacco/smoking, alcohol use (as applicable)
        ii. taking prescribed medications (access, affordability)
        iii. clinic visit
        iv. lab tests
    c. Next, we want to understand whether or not, COVID19 related lockdown influenced your livelihood/employment/income-either for you or your family members?
        i. How the COVID-19 related lockdown in your city affected children's education?
        ii. Please can you describe if you have experienced any sort of fear or stigma related to COVID-19?
3. Please can you suggest, how we can overcome some of these challenges: at individual-level, and society-level (if any)
4. List any two points that we can learn from this current situation: positive/negative for your family/society/state/country
5. What are two things that you will change in your life when the COVID-19 crisis is over?
6. Is there anything else that you want to discuss regarding COVID-19 impacts that we have not covered earlier?
